# Preferential expression of NY-BR-1 and GATA-3 in male breast cancer

**DOI:** 10.1007/s00432-017-2542-z

**Published:** 2017-11-07

**Authors:** Giovanni Battista Biserni, Enrico Di Oto, Linda Eszter Moskovszky, Maria Pia Foschini, Zsuzsanna Varga

**Affiliations:** 1 Unit of Anatomic Pathology “M. Malpighi”, Department of Biomedical and Neuromotor Sciences, University of Bologna, at Bellaria Hospital, Bologna, Italy; 2 Department of Pathology and Molecular Pathology, University Hospital Zurich, Schmelzbergstrasse 12, 8091 Zurich, Switzerland

**Keywords:** NY-BR-1, GATA-3, Primary male breast cancer, Metastatic male breast cancer

## Abstract

**Background:**

Male breast cancer is an uncommon disease often discovered in advanced stage; thus, in the setting of metastatic adenocarcinoma, breast origin must be taken to account. Breast markers as NY-BR-1, GATA-3, mammaglobin, and BRST-2 are established tools for labelling primary and metastatic female breast cancer; however, none of them has been sufficiently studied in male breast cancer. The aim of this study was to analyze the expression of these markers in male breast cancer.

**Materials and methods:**

Thirty consecutive cases of male breast cancer and eight loco-regional metastases were re-revaluated, assembled in tissue micro array (TMA), and stained with immunohistochemistry (IHC) for NY-BR-1, GATA-3, mammaglobin, and BRST-2. The IHC stains were scored either positive or negative. In addition, concordant expression patterns of primary tumors and matched metastasis were noted.

**Results:**

30 of 30 (100%) primary tumors and 8 of 8 (100%) metastases were positive for NY-BR-1. 30 of 30 (100%) primary tumors and 6 of 8 (75%) metastases were positive for GATA-3. 22 of 30 (73.3%) primary tumors and 6 of 8 (75%) metastases were positive for Mammaglobin. 18 of 30 (60%) primary tumors and 5 of 8 (62.5%) metastases were positive for BRST-2. Differences in staining percentage were not significant with Fisher’s exact test.

**Conclusion:**

We found a high sensitivity for all the markers analyzed. Moreover, the expression of NY-BR-1 and GATA-3 seemed the most effective for labelling male breast cancer in primary and metastatic setting.

## Introduction

In metastatic cancer, the knowledge of the site of origin is a key factor in patients’ management. Indeed, most of the therapies available rely on tissue-specific features and tumoral molecular characterization. On the other hand, the identification of the site of origin of a newly discovered metastatic adenocarcinoma, especially in case of poorly differentiated cancers, can represent a diagnostic dilemma. To identify the site of the primary tumor, several immunohistochemical approaches have been developed (Dennis et al. 2005; Rubin et al. 2001); ideally, each is aiming to reach the best specificity and sensitivity with the use of an ideal number of marker combination.

Male breast cancer is an uncommon disease, accounting for < 1% of all breast tumors. Due to its rarity and to the absence of a population-based screening, it is characterized by a consistent diagnostic delay and a more advanced stage at presentation than in female breast cancer. It is reported that from 7 to 30% of patients present with stage IV disease (Sanguinetti et al. 2016; Harlan et al. 2010; Fentiman et al. 2006) and 11% of these patients seek medical help merely for symptoms or evidences of extramammary dissemination.

NY-BR-1 is a novel marker of differentiation of the female mammary gland. The expression of this protein has been almost solely detected in breast tissue, at every level of cancer progression: 60–100% of primary lesions show positive staining. Metastasis are positive in approximately 50% of cases, with a concordance > 88–91% with the primary tumor (Jäger et al. 2007; Varga et al. 2006). Other markers of mammary differentiation such as GATA-3, mammaglobin, and BRST-2 (or gross cystic disease fluid protein 15) have been successfully employed in female breast cancer. These markers exhibit prognostic and therapeutic implications, represent important tools for detecting primary breast cancer and metastasis, and monitor lymph nodes during and after surgery (Wang et al. 2009; Kandalaft et al. 2016). Yet, none of these markers has been sufficiently investigated in male breast cancer.

The aim of this study was to analyze the expression of female breast cancer established markers NY-BR-1, GATA-3, mammaglobin, and BRST-2 in a cohort of male breast cancer in primary and metastatic lesions.

## Materials and methods

### Patients’ cohort

Thirty consecutive cases of primary male breast cancer were retrieved from the Department of Surgical Pathology and Molecular Pathology, University Hospital, Zürich, Switzerland. Paraffin embedded in formalin fixed tissues (FFPE) of the primary tumor and of normal breast tissues were available in all cases. In eight cases, axillary or distant metastases (7 to lymph nodes and 1 to the brain) were also available in FFPE blocks.

The 30 male breast cancer cases consisted of 23 invasive ductal carcinomas, no special type (NST), 4 papillary intracystic (with foci of invasion) carcinomas, and 2 micropapillary and 1 tubulo-lobular carcinomas. Four of thirty cases were grade 1, 21 of 30 grade 2, and 5 of 30 grade 3. The mean age of the patients at surgery was 66 (ranging from 39 to 92). All patients underwent mastectomy and received no systemic chemotherapy prior to surgery. Regional lymph nodes metastases were present in seven cases (1 grade 1, 4 grade 2, and 2 grade 3), and distant metastasis to the brain in one case of grade 3 tumor.

Estrogen receptor (ER) and progesterone receptor (PR) were re-assessed by immunohistochemistry (IHC), and Her2 status was detected with IHC and with fluorescent in situ hybridization (FISH).

Twenty-nine of thirty (96.6%) primary tumors were ER positive, and 26 of 30 cases (86.6%) PR positive. For ER, the percentage of positive cells ranged from 5 to 100%; for PR, positive cells represented from 3% to 100% of the total.

One of the 30 cases (3%) was Her2 positive (score 3 + and FISH amplified).

Lymph nodes and brain metastasis were always concordant in asset of hormone receptors and Her2 status with the matched primary tumors.

### Case selection

Cases were included in this study when paraffin blocks were available with adequate tissue to allow further analysis. Hematoxylin–eosin (HE)-stained slides of all cases were retrieved for histological evaluation. In case HE slides used for diagnosis were unavailable, new sections have been prepared. All male breast cancer cases underwent histological review and were classified and graded according to currently available criteria (Tavassoli and Eusebi 2009; Elston and Ellis 2002) by an experienced pathologist (ZV), and the most representative tumor blocks were selected for the study.

**Fig. 1:**
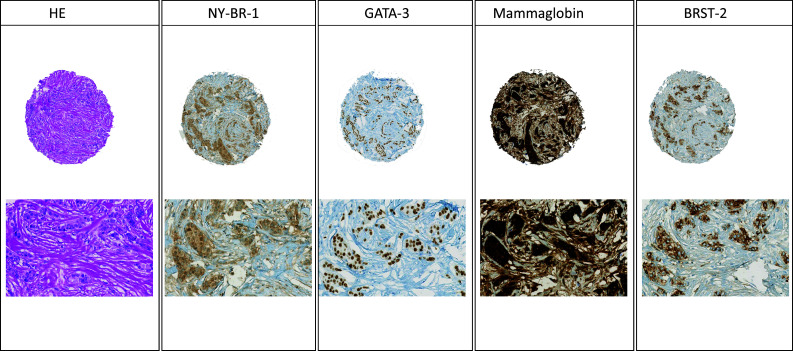
Morphology and immunohistochemical panel for male ductal (NST) breast cancer (low and high magnification) with NY-BR-1, GATA-3, mammaglobin, and BRST-2

Representative HE sections are shown in Fig. 1.

### Tissue microarray (TMA) construction

Invasive carcinomas both in the primary tumor and the metastatic deposits were identified on HE sections. In addition, areas of normal breast tissues were also selected on HE sections. Two cores of 0.6 mm each were punched from each area and transferred to an empty paraffin block under the guidance of a precision tool.

### Immunohistochemistry for NY-BR-1, GATA-3, mammaglobin, and BRST-2

The immunohistochemistry for NY-BR-1, GATA-3, mammaglobin, and BRST-2 have been conducted on the whole cohort with specific antibodies. Slides underwent pretreatment with TRIS/BORAT/EDTA Buffer and subsequently were treated with the antibodies on an automated benchmark (Ventana, Roche, Tucson, AZ, USA). Table 1 shows, in detail, laboratory protocol used for each antibody.

**Table 1:** Product characteristics and antibody-specific immunohistochemistry protocol

Name	NY-BR-1	GATA-3	Mammaglobin	BRST-2
Clone	NY-BR-1 + 2	L50-823	304-1A5	D6
Species	Mouse	Mouse	Mouse	Mouse
Dilution	1:100	1:250	1:200	1:4
Pretreatment	64 min	80 min	30 min	24 min
Manufacturer	Thermo scientific	Biocare medical	Biologo	Covance resp. BioLegend

To assess each protein expression, the tissue cores were scored by two pathologists (GBB, ZV) according to the percentage of cells showing a successful staining. The stains were acceptable as positive, if the internal control on the test breast tissue slide, respectively, the normal breast glands on the tissue cores were positive. The evaluation was conducted at least on 60 cells, alternatively on the maximal number assessable if less invasive tumor cells were available. Staining intensity was not considered. To classify each lesion as either positive or negative, the following scoring was made.

NY-BR-1: a tumor was considered positive, when at least a cluster of carcinoma cells exhibited staining in the cytoplasm or in the nucleus (Varga et al. 2006)
GATA-3: a case containing more than 5% of stained nuclei was considered positive for the expression of GATA-3 (Wendroth et al. 2015)

Mammaglobin and BRST-2: immuno-positivity was scored as follows: diffuse cytoplasmic staining in more than 1% of the population.

Concordant expression between primary tumors and matched metastasis was observed when both the lesions scored either positive or negative for the same marker.

### Statistical analysis

Fisher’s exact test was performed on the differences of the staining percentage of positive cases; a threshold of *p* < 0.05 was considered to reflect significant differences between groups.

## Results

### NY-BR-1

All 30 cases showed the expression of NY-BR-1 in primary lesions and in all 8 metastases.

In the primary tumors, sensitivity was 96.5% ranging from 90 to 100%; metastases showed 100% sensitivity in all the cases considered, and the expression was always concordant in with the matched primary tumor.

### GATA-3

All 30 primary tumors stained positively for GATA-3, whereas metastases were positive in six of eight cases (5 of 7 for lymph nodes and the brain metastasis).

Sensitivity in primary lesions was, on average, 93%, ranging from 50 to 100%, in metastases it was 96.6%, ranging from 90 to 100%.

### Mammaglobin

Twenty-two of thirty primary lesions (73.3%) scored positive for mammaglobin, with a sensitivity of 47%, on average, ranging from 2 to 100%. 6 metastases (5 of 7 for lymph nodes and the brain metastasis) showed positive staining for mammaglobin, sensitivity was 44.8% of the total, on average, and ranged from 4 to 100%. Concordance between primary and secondary lesion was observed in 7/8 cases.

### BRST-2

Eighteen of thirty (60%) primary tumors were positive for BRST-2; the sensitivity varied from 2 to 100% and was on average 25.3%. Five of eight cases of corresponding metastasis were positive (4 of 7 to lymph nodes and the brain lesion); the sensitivity was 59%, ranging from 10 to 100. In 4/8 cases, the expression of this marker was concordant between primary tumor and metastasis.

Results are summarized in Table 2 and representative immunohistochemistry stains are also shown in Fig. 1.

**Table 2:** Sensitivity of breast markers in concordant primary tumors and matched metastases; in brackets, percentage of the total

	NY-BR-1	GATA-3	Mammaglobin	BRST-2
Positive cases primary tumor	30 of 30 (100%)	30 of 30 (100%)	22 of 30 (73.3%)	18 of 30 (60%)
Positive cases metastasis	8 of 8 (100%)	6 of 8 (75%)	6 of 8 (75%)	5 of 8 (62.5%)
Concordance primary tumor vs. metastasis	8 of 8 (100%)	6 of 8 (75%)	7 of 8 (87.5%)	4 of 8 (50%)

Each is referred to the marker of the heading

### Statistical analysis

Differences in staining percentage were not significant with Fisher’s exact test.

## Discussion

As male breast cancer is often diagnosed at an advanced stage, breast origin must be taken into account in case of newly discovered metastatic adenocarcinomas. In this study, we could show that immunohistochemical profile of the so-called breast markers can be reliably applied also to male breast cancer both in the primary and in the metastatic settings.

In this study, we observed a high sensitivity for all known female breast markers in a cohort of male breast cancer and in their corresponding axillary and/or distant metastases. Among the markers, NY-BR-1 and GATA-3 showed the highest expression.

NY-BR-1 is frequently expressed in normal breast, female breast cancer, and metastasis, whilst no significant expression has been detected in any other normal or neoplastic tissue (Varga et al. 2006). The highest sensitivity rate for NY-BR-1 was observed in normal glandular breast tissue and in ductal carcinoma in situ in females (Varga et al. 2006). Invasive female breast carcinomas were shown to express NY-BR-1 depending on histological grading, being mostly expressed in G1 and G2 carcinomas (Varga et al. 2006). Therefore, NY-BR-1 is considered both as a breast-specific marker and as a differentiation marker as well (Varga et al. 2006). Since our cohort comprised a significant number of grade 1 and 2 primary tumors (25/30) and metastases came mainly from neoplasms of the same grade of differentiation (5/8), positive cases for NY-BR-1 were likely to represent the vast majority in this study. As NY-BR-1 was shown to be inversely related to breast differentiation, problems may arise in clinical practice when considering distant metastasis from a poorly differentiated carcinoma. Assumed the peculiar specificity of NY-BR-1, it is to emphasize that high-grade lesions may lose the expression of the protein more frequently than reported in the present study. Nevertheless, the concordance highlighted between metastases and matched primary tumors (100%) encourages the application of this marker also in metastatic setting.

GATA-3 is a protein linked to estrogen receptor pathway. It is not surprising that we found a high prevalence of positive cases in a group with 96.6% of ER + tumors. Male breast cancer is expected to be ER + in a higher percentage of case rather than female breast cancer (Doebar et al. 2017).

The role of GATA-3 has been extensively investigated in female breast pathology.

Sangoi et al. compared the sensitivity of GATA-3 to mammaglobin and GCDFP-15 (BRST-2), and found that GATA-3 showed superior sensitivity in both metastatic carcinoma and matched primary tumor. Moreover, IHC for GATA-3 harbored consistently fewer problems of interpretation, thanks to less background staining, than the other two markers (Sangoi et al. 2016).

In our subset of carcinomas, we observed the same tendency. The sensitivity of mammaglobin vs GATA-3 in labelling male breast cancer was 73.3 vs 100%, and BRST-2 vs GATA-3 was 60 vs 100%, respectively. Expression in metastases showed similar levels of concordance: 75% for both GATA-3 and mammaglobin, and 62.5% for BRST-2.

Although GATA-3 has been reported to be highly sensitive for breast tumors, it is not entirely specific. Commonly, GATA-3 expression characterizes tumors arising from skin adnexa, urothelium, salivary glands, and pancreatic ducts, whereas adenocarcinomas of lung, stomach, colon, and prostate showed positivity to a lesser extent (Miettinen et al. 2014).

To avoid clinical concerns arising from the lack of specificity of GATA-3 in the setting of metastatic adenocarcinoma, we carried further analysis on the same cohort with the novel marker NY-BR-1, which showed a high concordance between GATA-3 and NY-BR-1.

Mammaglobin, is a protein of unknown function, whose expression increases tenfold in breast cancer (Watson and Fleming 1996). It has not been detected in any other type of cancer, whereas in small amounts only in normal breast tissue.

The discovery of its specificity brought to employ this protein for labelling breast neoplasms. The expression varies between different subtypes: high in luminal and Her2 positive tumors and rather low in triple negative and basal-like tumors. Mammaglobin raised sensitivity and specificity in distinguishing between cutaneous metastases of breast carcinomas and sweat gland carcinomas (Lewis et al. 2011; Diel et al. 1996). Other studies conducted with RT-PCR analysis reported mammaglobin utility in monitoring lymph nodes, predicting the risk of metastases, and characterizing pleural effusions in breast cancer patients (Ciampa et al. 2004).

However, given its high specificity, IHC for mammaglobin in our study showed a sensitivity of 73.3%. These data make this marker only partially suitable for labelling male lesions, if adopted on its own.

BRST-2 (also known as gross cystic disease fluid protein GCDFP-15) is a breast marker expressed in up to 73% of female breast cancer (Fritzsche et al. 2007). His detection in specimens is clearly influenced by the histological and molecular subtype: sensitivity is high in tumors with apocrine and lobular signet ring features, as well as androgen receptor positivity which is also related to BRST-2 expression. On the other hand, medullary histological phenotype and triple negative intrinsic subtype showed lower rates of BRST-2 positivity.

Similar to GATA-3, this marker does not exclusively label breast cancer: it is expressed by apocrine glands, including those in the breast, the tracheobronchial tree, sweat gland carcinomas, and salivary gland carcinomas (Wick et al. 1998). Finally, an important overlap is observed in 5–6% of lung adenocarcinomas. In our cohort, we found that 60% of male breast cancer scored positive for BRST-2, enhancing a substantial lack of sensitivity.

Either in female or in male breast cancer, BRST-2 and mammaglobin may show a clinical value only when combined with other markers. In our study, we could show that NY-BR-1 possesses greater sensitivity than both the aforementioned markers.

In the setting of metastases of unknown origin, there are established immunohistochemical panels to apply. In case clinical history, complete physical examination, routine laboratory tests, imaging, and radio-metabolic techniques do not come to any conclusive results, the detection of tumor specific antigens or specific immunohistochemical stains can be also helpful.

For a subset of tumors, one marker alone may show the sufficient diagnostic accuracy.

In appropriate clinical context, the prostate-specific antigen (PSA) for prostate cancer, PAX8 for ovarian cancer, alphafetoprotein for liver cancer, and TTF1 for lung cancer can be reliably used as a single marker (Dennis et al. 2005; Rubin et al. 2001; Woodard et al. 2011).

If one marker alone does not possess sufficient specificity nor sensitivity for the suspected neoplasms, a combination of IHC stains may provide additional support. An initial approach with cytokeratin subtypes as CK7 and CK20 gives a first hint at the likely site of origin for carcinomas as to gastrointestinal or gynecological origin. These tests can be completed with CDX2 and CK19 for upper gastrointestinal tract, biliary ducts and pancreas, thyroglobulin for thyroid adenocarcinoma, NY-BR-1 and GATA-3 for female breast cancer, Mel-A, S100 and HMB45 for melanoma, and vimentin and Mel-A for kidney and adrenal gland tumors.

In our study we could show that NY-BR-1 and GATA-3 are highly sensitive breast markers in primary and metastatic male breast cancer. As already demonstrated in females, both NY-BR-1 and GATA-3 expression in male breast cancer outperforms the use of mammaglobin and BRST-2 alone. Using either NY-BR-1 or GATA-3, rather than mammaglobin and BRST-2 may help confirming breast origin both in primary and in metastatic carcinoma in males.
